# Polydatin Alleviates Diabetes-Induced Hyposalivation through Anti-Glycation Activity in db/db Mouse

**DOI:** 10.3390/pharmaceutics14010051

**Published:** 2021-12-27

**Authors:** Hyung Rae Kim, Woo Kwon Jung, Su-Bin Park, Hwa Young Ryu, Yong Hwan Kim, Junghyun Kim

**Affiliations:** Department of Oral Pathology, School of Dentistry, Jeonbuk National University, Jeonju 54896, Korea; rlagudfo31@gmail.com (H.R.K.); wkjungjbnu@gmail.com (W.K.J.); tnqls309@gmail.com (S.-B.P.); naive17jbnu@gmail.com (H.Y.R.); kg7229ku@gmail.com (Y.H.K.)

**Keywords:** advanced glycation end products, diabetes, polydatin, salivary gland

## Abstract

Polydatin (resveratrol-3-O-β-mono-D-glucoside) is a polyphenol that can be easily accessed from peanuts, grapes, and red wine, and is known to have antiglycation, antioxidant, and anti-inflammatory effects. Diabetes mellitus is a very common disease, and diabetic complications are very common complications. The dry mouth symptom is one of the most common oral complaints in patients with diabetes mellitus. Diabetes mellitus is thought to promote hyposalivation. In this study, we aimed to investigate the improvement effect of polydatin on diabetes-induced hyposalivation in db/db mouse model of type 2 diabetes. We examined salivary flow rate, TUNEL assay, PAS staining, and immunohistochemical staining for AGEs, RAGE, HMGB1, 8-OHdG, and AQP5 to evaluate the efficacy of polydatin in the submandibular salivary gland. Diabetic db/db mice had a decreased salivary flow rate and salivary gland weight. The salivary gland of the vehicle-treated db/db mice showed an increased apoptotic cell injury. The AGEs were highly accumulated, and its receptor, RAGE expression was also enhanced. Expressions of HMGB1, an oxidative cell damage marker, and 8-OHdG, an oxidative DNA damage marker, increased greatly. However, polydatin ameliorated this hypofunction of the salivary gland and inhibited diabetes-related salivary cell injury. Furthermore, polydatin improved mucin accumulation, which is used as a damage marker for salivary gland acinar cells, and decreased expression of water channel AQP5 was improved by polydatin. In conclusion, polydatin has a potent protective effect on diabetes-related salivary gland hypofunction through its antioxidant and anti-glycation activities, and its AQP5 upregulation. This result suggests the possibility of the use of polydatin as a therapeutic drug to improve hyposalivation caused by diabetes.

## 1. Introduction

Most Saliva is a mixture of fluids secreted by the three major glands (submandibular, sublingual, and parotid) and several minor glands, containing 99% water; electrolytes such as potassium, magnesium, calcium, sodium, and immunoglobulins; enzymes; etc., and protects the oral cavity [1]. It is also necessary for the initiation of digestion and absorption of food and for maintaining the pH, tooth mineralization, oral microbiome, moist oral mucosal surfaces, and infection prevention of teeth and mucosa [2,3]. Therefore, proper salivation is essential for maintaining the oral environment.

Xerostomia is a disease that decreases salivation, can occur even if an objective decrease in salivation is not observed, can be triggered by a change in the ingredients of the saliva, and can be caused by radiotherapy, Sjögren’s syndrome, pathologies of the salivary glands, or medications [1,4]. Xerostomia causes diseases such as enamel demineralization, rampant decay, and super-infections by fungal diseases, is associated with oral dryness and decreased or altered taste and lowers the overall quality of life by negatively affecting swallowing and ingestion [5,6].

Diabetes mellitus (DM) is divided into type 1 and prevalent type 2, with its symptoms appearing slowly after the age of 40, which triggers a chain reaction of increased protein glycation due to high blood glucose, causes functional damage generated in many organ systems by promoting the creation of advanced glycation end products (AGEs), and results in ultimate complications such as cardiovascular disease, nephropathy, and retinopathy [7,8,9]. Oral manifestations due to DM include xerostomia, dental caries, periodontal disease, burning mouth, and poor wound healing, with xerostomia being a very common phenomenon, which 34% to 51% of diabetic patients experience [10,11,12].

Salivary stimulants such as chewing gum, vitamin C, malic acid, and pilocarpine or salivary substitutes are solutions for xerostomia, which only provide temporary symptom relief [13]. Additionally, there are some medications that cause xerostomia among the medications for the treatment of some existing diseases [14], and there are cases in which xerostomia is caused by xerostomia-containing drugs or the synergistic effect of the drugs if the person takes multiple medications as they get older [15]. Therefore, there is a need for research on therapeutic drugs that can simultaneously improve xerostomia in addition to alleviating existing diseases.

The rhizome and root of *Polygonum cuspidatum* are widely used as traditional herbal medicines for analgesic, diuretic, antipyretic, and expectorant purposes, its main active ingredient being polydatin (resveratrol-3-O-β-mono-D-glucoside) [16]. Polydatin exists in nature in the form of resveratrol and is abundantly present in peanuts, grapes, chocolates, and red wine [17]. Polydatin is known to have anti-diabetic, antioxidant, anti-inflammation, anticancer, and cardiovascular protective effects [18]. In addition, resveratrol is involved in the recovery process in the experimental irradiation-induced xerostomia in vivo model [19,20], and there are studies about resveratrol’s improvement on salivary dysfunction in a non-obese diabetic (NOD) mice model [21]. However, there are no studies about polydatin related to salivary secretion.

Therefore, this study aims to investigate the improvement effect of diabetes-induced salivary gland hyposalivation by polydatin in type 2 diabetic db/db mice.

## 2. Materials and Methods

### 2.1. Chemicals

Polydatin, with purity greater than 95%, was obtained from Sigma-Aldrich (St. Louis, MA, USA).

### 2.2. In Vivo Experiment Design in Normoglycemic Mice

To determine the oral dose of polydatin for in vivo experiment, 5-week-old male C57BL/6 mice were purchased from Damul Science (Daejeon, Korea). The mice were adapted to the environment for 1 week and then randomly divided into four groups: normal control (*n* = 5), polydatin 25 mg/kg (PD-25, *n* = 5), polydatin 50 mg/kg (PD-50, *n* = 5), polydatin 100 mg/kg (PD-100, *n* = 5). Polydatin was dissolved in 0.5% (*w/v*) sodium carboxymethylcellulose (Sigma-Aldrich, St. Louis, MA, USA). Polydatin was administered orally to mice on a daily basis for 3 weeks.

### 2.3. In Vivo Experiment Design in Hyperglycemic Mice

Fourteen male db/db leptin receptor-deficient type 2 diabetic mice aged 5 weeks and seven male wild type C57BL/6 mice aged 5 weeks were supplied by Central Lab Animal Inc. (Seoul, Korea). The mice were adapted to the environment for 1 week and then randomly divided into 3 groups based on the weight and fasting blood glucose levels as follows: normal group (Normal, *n* = 7), diabetic group (DM, *n* = 7), polydatin 100 mg/kg treated group (DM + PD, *n* = 7). Polydatin was dissolved in 0.5% (*w/v*) sodium carboxymethylcellulose and orally administered for 5 weeks. The animals were housed in a barrier system controlled by temperature (20–25 °C) and humidity (40–70%), with a 12:12 h light and dark cycle. Animal experiments were carried out according to procedures approved by the Institutional Animal Care and Use Committee of the Jeonbuk National University Laboratory Animal Center (IACUC, Approved No.: JBNU 2021-087).

### 2.4. Collection of Saliva

Mice were anesthetized with an intraperitoneal injection of ketamine at a dose of 80 mg/kg. Salivation was induced with an intraperitoneal injection of pilocarpine (Sigma-Aldrich, St. Louis, MA, USA) at a dose of 0.8 mg/kg. The volume of saliva was measured for 10 min after injection of pilocarpine using cotton balls. Cotton balls were kept in the oral cavity for 10 min to absorb saliva and were then immediately weighed on an electronic balance. Total saliva was calculated from the difference of pre and post-collection cotton ball weight in milligrams and converted to microliters. The salivary flow rate was calculated as microliters per minute.

### 2.5. Hematoxylin and Eosin (H&E) Staining

At necropsy, submandibular gland tissues were isolated. Formalin-fixed tissues were embedded in paraffin and sliced into 4 μm thick sections. The sections were deparaffinized and rehydrated using standard techniques and stained with hematoxylin solution gill no. 3 and eosin. The stained sections were dehydrated and cleared with xylene, before being observed under a light microscope.

### 2.6. Immunohistochemical Staining

The tissue sections were deparaffinized and rehydrated using standard techniques. The sections were incubated with 3% H_2_O_2_ for 10 min at room temperature (RT). They were then washed with 1X TBST buffer for 10 min at RT. Primary antibodies were diluted in 2.5% normal horse serum (Vector Laboratory, Bulingame, CA, USA). The primary antibodies were AGE (TransGenic, Kyoto, Japan), the receptor for AGE (RAGE, Santa Cruz, CA, USA), high mobility group box 1 (HMGB1, Abcam, Boston, MA, USA), 8-hydroxydeoxyguanosine (8-OHdG, Abcam, Boston, MA, USA), and aquaporin5 (AQP5, Bioworld Technology, St. Louis Park, MN, USA). Sections were visualized with the VECTASTAIN Elite ABC Universal Kit (Vector Laboratory, Bulingame, CA, USA). The staining was observed using a BX51 light microscope (Olympus, Tokyo, Japan). Ten unique fields of view were randomly selected in each slice at 100× magnification. Image-Pro software (Media Cybernetics, Rockville, MD, USA) was used for semi-quantitative analysis of the intensity of the immunohistochemical positive signal. The average optical density per unit area (mm^2^) was calculated for the relative amount of protein.

### 2.7. Oxidative Stress Assay in Salivary Gland

Frozen salivary gland tissues were homogenized in lysis buffer (150 mM NaCl, 1% Triton X-100 and 10 mM Tris, pH 7.4) containing protease inhibitor. The homogenate was centrifuged at 10,000× *g* for 10 min at 4 °C and the supernatant was collected for measurement of reactive oxygen species (ROS) levels. ROS levels were examined using a Mouse ROS ELISA Kit (MyBioSource, San Diego, CA, USA) according to the manufacturer’s instructions.

### 2.8. Periodic Acid-Schiff (PAS) Staining

The tissue sections were deparaffinized and rehydrated using standard techniques and oxidized in 1% periodic acid solution for 5 min and rinsed in distilled water. The sections were placed in Schiff reagent for 15 min and counterstained with hematoxylin.

### 2.9. TUNEL Assay

Apoptotic cells in the tissue were detected by an in situ cell death detection kit (Roche, Mannheim, Germany), according to the manufacturer’s instructions. The numbers of TUNEL-positive cells were counted under a fluorescence microscope (BX51, Olympus, Tokyo, Japan). Ten fields at 100× magnification were photographed randomly from each salivary gland with a fluorescence microscope. The total number of TUNEL-positive cells per unit area (mm^2^) was counted in each field using image analysis software (Image-Pro, Media Cybernetics, Rockville, MD, USA).

### 2.10. Statistical Analysis

Results are presented as mean ± standard deviation. Significant differences between groups were determined using one-way analysis of variance (ANOVA), followed by Tukey’s multiple comparison test. Differences with a *p*-value lower than 0.05 were considered to represent significant differences.

## 3. Results

### 3.1. Increased Salivation in Normoglycemic Mice by Polydatin and Selection of the Oral Dose of Polydatin

**Figure 1:**
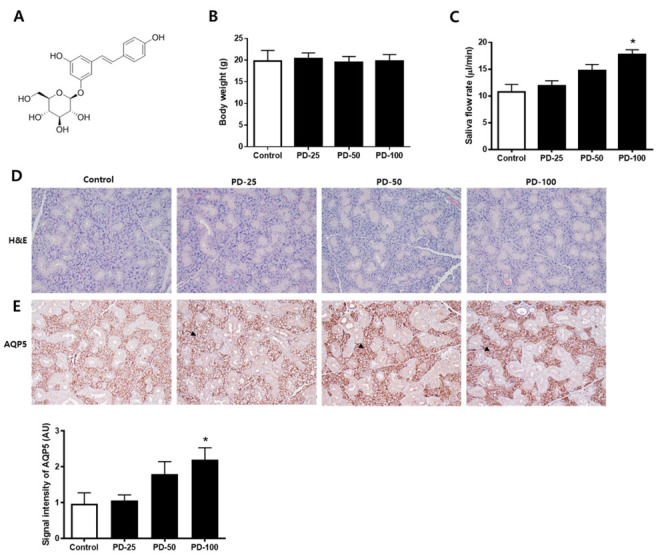
Effect of polydatin in the normoglycemic mice. (**A**) Chemical structure of polydatin. (**B**) Body weight. (**C**) Salivary flow rate. (**D**) Representative submandibular glands were stained with H&E. (**E**) Immunohistochemical staining for AQP5. Quantification of the AQP5 signal intensity. Arrows indicate AQP5 positively stained cells. The values in the bar graphs represent the means ± SD, *n* = 5. * *p* < 0.05 vs. normal control group.

Changes in salivation by polydatin (Figure 1A) were checked in normoglycemic mice. First, it was confirmed that there was no significant change in body weight (Figure 1B). In addition, it was confirmed that salvation increased depending on the concentration after administration of polydatin for 3 weeks (Figure 1C). H&E staining was performed to confirm histological changes and no compound-related histopathological changes were found (Figure 1D). In addition, the expression level of AQP5, a water channel protein important for salivation, was increased by polydatin treatment (Figure 1E). Therefore, 100 mg/kg of polydatin was selected as the administration concentration for the in vivo test using hyperglycemic db/db mice.

### 3.2. Polydatin Improves Hyposalivation in Type 2 Diabetic Mice

**Figure 2:**
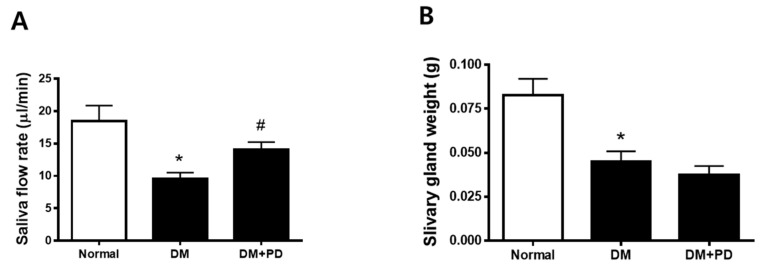
Effect of polydatin on salivation in type 2 diabetic db/db mouse. (**A**) Salivary flow rate. (**B**) Submandibular gland weight. Data are presented as the mean ± standard deviation (*n* = 7). * *p* < 0.05 vs. Normal group; # *p* < 0.05 vs. DM group.

At the endpoint, the body weight was higher in the DM group with no difference between the DM + PD groups. Additionally, blood glucose levels also increased in the DM group with no difference between the DM + PD groups (Table 1). As a result of the measurement of salivation, it was confirmed that salivation decreased significantly in the DM group and increased in the DM + PD groups (Figure 2A). The weight of the salivary gland decreased significantly in the DM group with no difference between the DM + PD groups (Figure 2B).

### 3.3. Polydatin Inhibits Apoptosis in the Submandibular Gland in Type 2 Diabetic Mice

**Figure 3:**
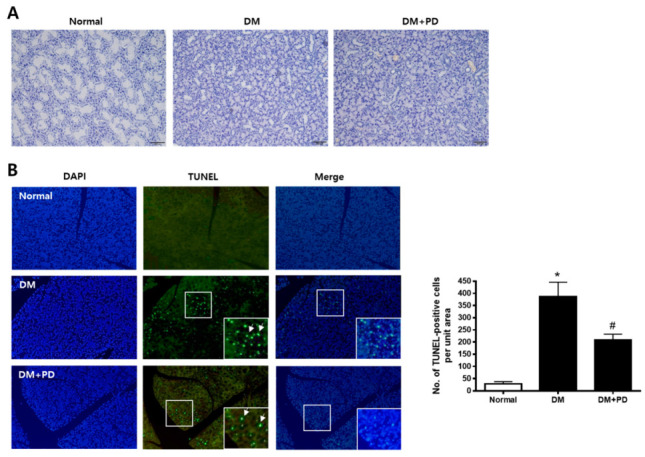
Effect of polydatin on histological changes and apoptosis in submandibular glands. (**A**) Histological analysis of the submandibular gland was performed and images of H&E staining are presented. (**B**) TUNEL staining is presented. Arrows indicate TUNEL-positive cells. Data are presented as the mean ± standard deviation (*n* = 7). * *p* < 0.05 vs. Normal group; # *p* < 0.05 vs. DM group.

H&E staining was conducted to evaluate the histological change of the submandibular gland. As shown in Figure 3A, the size of duct markedly decreased in the DM group with no difference between the DM + PD groups. To confirm whether this histological change is due to apoptotic injury, a TUNEL assay was conducted in the submandibular gland. The number of TUNEL-positive cells increased significantly in the DM group and decreased significantly in the DM + PD groups (Figure 3B).

### 3.4. Polydatin Decreases AGEs Accumulations and RAGE Expression in the Submandibular Gland in Type 2 Diabetic Mice

**Figure 4:**
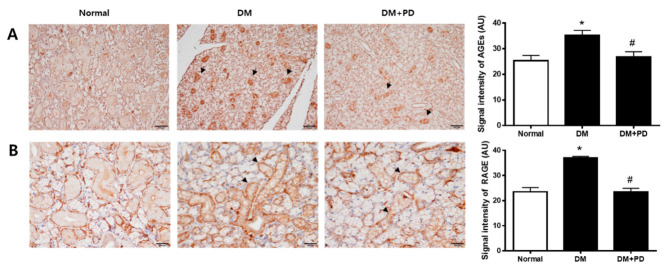
Effect of polydatin on the expression of AGEs and RAGE in submandibular glands. (**A**) Immunohistochemical staining for AGEs. Quantification of the AGEs signal intensity. Arrows indicate AGEs positively stained cells. (**B**) Immunohistochemical staining for RAGE. Quantification of the RAGE signal intensity. Arrowheads indicate RAGE positively stained cells. Data are presented as the mean ± standard deviation (*n* = 7). * *p* < 0.05 vs. Normal group; # *p* < 0.05 vs. DM group.

In hyperglycemia, an increase in the glycation process accelerates the accumulation of AGEs [22]. Therefore, the measurement of AGEs in the submandibular gland confirmed the accumulation of AGEs in the DM group and the reduction of AGEs by polydatin treatment (Figure 4A). As for RAGE, the expression level of RAGE increases due to hyperglycemia, resulting in the generation increase in ROS [23]. Therefore, the result of checking the expression level of RAGE confirmed that the expression of RAGE was increased in the DM group and decreased in the DM + PD groups treated with polydatin (Figure 4B).

### 3.5. Polydatin Decreases the Expressions of HMGB1 and 8-OHdG in the Submandibular Gland in Type 2 Diabetic Mice

**Figure 5:**
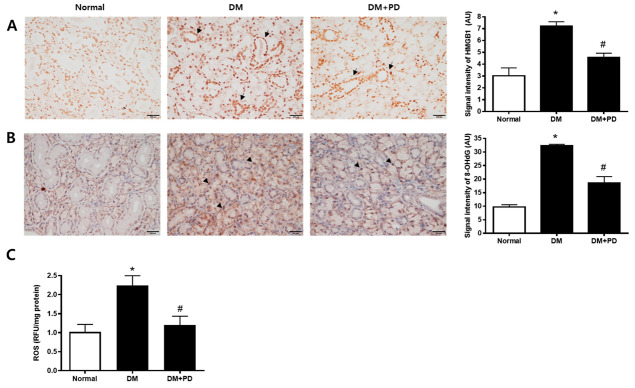
Effect of polydatin on ROS generation in submandibular glands. (**A**) Immunohistochemical staining for HMGB1. Quantification of the HMGB1 signal intensity. Arrows indicate HMGB1 positively stained cells. (**B**) Immunohistochemical staining for 8-OHdG. Quantification of the 8-OHdG signal intensity. Arrowheads indicate 8-OHdG positively stained cells. (**C**) ELISA assay for ROS in the salivary gland. Data are presented as the mean ± standard deviation (*n* = 7). * *p* < 0.05 vs. Normal group; # *p* < 0.05 vs. DM group.

To confirm the change caused by oxidative stress-induced cell damage, the accumulation pattern of HMGB1 was checked [24]. In the DM group, the accumulation of HMGB1 and accumulation reduction due to polydatin treatment were confirmed (Figure 5A). To evaluate salivary ROS levels in d-galactose-induced aging rats, we examined ROS ELISA assay and immunohistochemical staining of 8-OHdG, a marker for oxidative DNA damage [25], in salivary gland tissues. As shown in Figure 5B,C, ROS generation and 8-OHdG expression were largely increased in the DM group. Polydatin significantly prevented the generation of ROS and oxidative DNA damage in diabetic mice.

### 3.6. Polydatin Decreases Mucin Accumulation and the Increased AQP5 Channel in the Submandibular Gland in Type 2 Diabetic Mice

**Figure 6:**
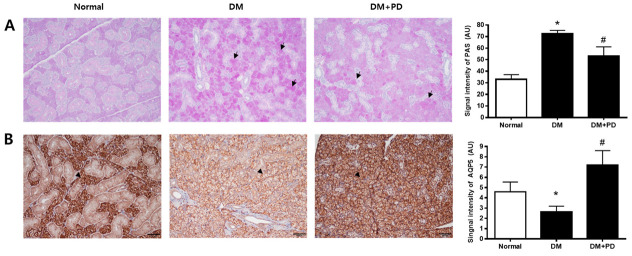
Effect of polydatin on mucin accumulation and AQP5 expression in submandibular glands. (**A**) Periodic acid-Schiff (PAS) staining of submandibular glands. Arrows indicate PAS positively stained cells. (**B**) Immunohistochemical staining for AQP5. Quantification of the AQP5 signal intensity. Arrowheads indicate AQP5 positively stained cells. Data are presented as the mean ± standard deviation (*n* = 7). * *p* < 0.05 vs. Normal group; # *p* < 0.05 vs. DM group.

The change aspect of mucin accumulation in salivary gland acinar cells was confirmed through Periodic acid-Schiff (PAS) staining. In the DM group, it was possible to confirm the increase in mucin accumulation and that it decreased due to polydatin treatment (Figure 6A). In addition, the expression level of AQP5 was significantly reduced in the DM group and increased in the DM + PD groups by polydatin treatment (Figure 6B).

## 4. Discussion

Hyperglycemia due to high blood glucose levels is a characteristic of diabetes, which causes many changes in the body through damage to various organs. Hyposalivation and submandibular gland dysfunction have been reported in in vivo models, especially in type 2 diabetic mice [26], non-obese diabetic mice [27], streptozotocin (STZ)-induced type 1 diabetic rats [28], and this study confirmed the improvement effect of polydatin on diabetes-induced hyposalivation in a type 2 diabetic db/db mice model.

This study could not confirm the glycemic control effect of polydatin. Previous studies have confirmed a decrease in glucose in a STZ-induced diabetic mice model [29,30]. A decrease in blood glucose was also confirmed in STZ-induced diabetic male C57BL/6J mice after 8 weeks of polydatin administration [31]. In addition, similar to this study, there is a study showing that blood glucose decreases when 100 mg/kg of polydatin is administered in type 2 diabetic db/db female mice for 4 weeks [18,32]. However, in the oral administration experiment of 100 mg/kg polydatin for 4 weeks in type 2 diabetic male C57BL/6J mice via a high-fat diet, the reduction of blood glucose could not be confirmed [33]. These results are thought to have resulted from the combination of female and male insulin resistance difference [34,35], the difference in pathogenesis for type 1 and 2 diabetes mellitus [36], and the usage, which requires a more detailed study to find the causes. However, in this study, it was possible to confirm the improvement of salivary gland hyposalivation by polydatin without the alleviation of hyperglycemia, which is considered to be a significant finding since there are therapeutic drugs that show a protective effect of diabetic complications without glycemic control [37].

The level of ROS maintained by the antioxidant system of cells in the body increases due to hyperglycemia, disrupting the balance; increased ROS causes apoptosis and inflammation through various signaling paths in cell compositions such as proteins and lipids and organ systems such as the brain, blood vessels, and kidneys, ultimately destroying the systems [38,39,40].

In addition, the Maillard reaction (non-enzymatic) initiated by the glycation of amino groups and carbonyl groups of proteins causes molecular glycation in circulating proteins, including albumin, lipoprotein, and insulin, and allows for the easy generation of ROS through increased oxidation sensitivity, resulting in the increase in the apoptosis pathway. Hyperglycemia promotes the generation of a substance called AGEs, the final form of products produced by glycation; AGEs produce irreversible cross-links between proteins, transform properties and structure, increase tissue fibrosis and stiffness, cause organic dysfunction, and combine with RAGE to increase oxidative stress and pro-inflammatory signaling pathways. Consequently, it induces vascular complications such as diabetic atherosclerosis, retinopathy, diabetic nephropathy, neuropathy, and impaired wound healing [41,42,43]. In type 2 DM, hyperglycemia induces salivary gland cell death through the generation of ROS, leading to xerostomia [44], and there is a study in which an increase in RAGE was confirmed in the salivary gland of patients with Sjögren’s syndrome [45].

In this study, the accumulation of AGEs and the increased expression of RAGE in the DM group showed that the increase in glycation caused by hyperglycemia was related to salivary gland hyposalivation. There are reports about the accumulation inhibition of AGEs by resveratrol [46], the formation inhibition of polydatin’s BSA-MGO reaction induced AGEs [47], and the expression inhibition of RAGE in Schwann cells of diabetic rats [48], and this study confirmed the reduction of AGEs and RAGE by polydatin in the DM + PD group, showing in addition that the improvement of diabetes-induced hyposalivation by polydatin treatment is related to the anti-glycation effect of polydatin.

HMGB1 is a chromatin-binding nuclear protein that acts as an extracellular signal in damaged cells, is related to oxidative stress, and is released in apoptosis [24,49]. In addition, there is a report that the accumulation of HMGB1 is observed in live cells with oxidative-induced damage [50]. There is another report that 8-OHdG is a biomarker of DNA-endogenous oxidative damage, which is mainly used as an indicator of oxidative stress in diabetic nephropathy [25,51]. HMGB1 increased in the submandibular gland of the diabetes-xerostomia rats model [52], and 8-OHdG increased in the saliva of xerostomia patients [53]. In this study, the result of checking DNA damage caused by a hyperglycemia-induced ROS increase in the DM group confirmed the increase in oxidative DNA damage of salivary gland cells by oxidative stress through the increase in HMGB1 and 8-OHdG. This study confirmed that glycation and oxidative stress markers increased by hyperglycemia were decreased by polydatin in the submandibular gland of type 2 diabetic mice, which confirmed that polydatin has antioxidative and anti-glycation effects.

Resveratrol, commonly found in nature in things such as grapes, peanuts, wine, and chocolate products, is a type of polyphenol and is known to have various pharmacological effects, such as anti-inflammation, antioxidation, and anti-tumor effects [54]. There is a report that polydatin, a form in which glucose is combined with resveratrol, is the richest form of resveratrol and metabolizes with resveratrol, which has several pharmacological effects when absorbed in the body, has a higher antioxidation and anti-glycation effect based on better absorption, and has better metabolic stability than resveratrol when administered orally with the same dose as resveratrol [17,47,55,56]. It can be seen that these pharmacological activities of polydatin were involved in the inhibition of hyposalivation and apoptosis in db/db mice.

In the present study, the effective dose of polydatin is 100 mg/kg. Considering an average body weight of an adult of 60 kg, this dose for a 60 kg human is equal to 0.4 g/day. The recommended dose of polydatin as a human food supplement is 160 mg/day [57]. Even if a high dose of polydatin was required to obtain a significant effect in salivary gland dysfunction, polydatin is known to be absent of side effects and toxicity.

Mucin, the main component of saliva, can change in the pathological condition of the salivary gland. In addition, the over-expression of mucin is promoted by pro-inflammatory cytokines, and cytokine production is associated with an increase in ROS [58,59,60]. Huang et al. reported that db/db mice demonstrated that PAS-positive mucins accumulated in the acini of submandibular glands [58]. Mucin accumulation in salivary glands also suggests that the salivary secretory function was decreased under hyperglycemic conditions. Therefore, as a result of measuring submandibular gland mucin through PAS staining, the damage of salivary gland acinar cells caused by diabetes in the DM group was confirmed through an increase in mucin. The prevention of salivary gland dysfunction by the antioxidant effect of polydatin was confirmed through a decrease in mucin in the DM + PD groups

Saliva is produced and secreted by the acinar cells and duct cells of the salivary gland with its flow initiated by an increase and release of Ca^2+^ in the acinar cells, which in turn creates osmotic pressure, and fluid is secreted by the AQP5 channel in the membrane by the osmotic gradient [61]. AQP is present in most organisms, including mammals, plants, and other microbes. There are 13 AQP gene families in humans, which function as water channels in the eyes, brain, and secretory glands [62]. Among them, AQP5, which is localized to the apical membrane of the salivary gland acinar cells, plays a very important role in the generation of tears, saliva, and exocrine secretions [63], and mice with AQP5 knockout have salivation reduced by more than 60% compared to normal mice [64]. As a result of checking the expression of AQP5, a significant decrease was confirmed, which is presumed to be related to the decrease in AQP5 expression due to the increase in ROS [65], and it is assumed that the reduction of AQP5 expression was alleviated by the antioxidation effect of polydatin.

In the present study, polydatin also promoted saliva secretion in normoglycemic mice. we did not clearly show the mode-of-action of polydatin on salvation in normal mice. However, polydatin is a natural precursor of resveratrol. Polydatin showed better oral absorption and metabolic stability than resveratrol since its serum concentration was 3–4 times higher after oral administration at the same dosage [55]. Polydatin is structurally the same as resveratrol except that it has a glucoside group attached to the C-3 position in place of a hydroxyl group. This substitution makes polydatin more water-soluble and resistant to enzymatic breakdown than resveratrol [16]. Polydatin shares resveratrol’s beneficial pharmaceutical properties, with the further advantage of being more abundant than resveratrol [66]. Multiple direct targets of resveratrol, including COX, PPAR, eNOS, and Sirt1, have been identified. Inoue et al. reported that resveratrol increased Sirt1 activity in the salivary gland of NOD mice [21]. This protein regulates a wide range of cellular processes. Forkhead box O (FOXO) transcription factors have been identified as substrates of SIRT1 [67]. SIRT1 regulates the transactivation activity of FOXO by catalyzing its deacetylation [68]. It was recently reported that FOXO1 directly regulates AQP5 expression in salivary gland acinar cells. FOXO1 is a direct regulator of AQP5 expression in salivary gland acinar cells through its interaction with the promoter region of AQP5 [69]. AQP5 is the most important protein for salivation since it directly mediates the transcellular movement of water from the basal to apical or lumen side of acinar cells [70]. Based on the results, we have suggested a possibility that polydatin may promote salivation via the upregulation of AQP5 expression. To identify this additional role of polydatin on AQP5 expression, we performed the immunohistochemical staining for AQP5 in salivary glands. AQP5 was highly expressed in acinar cells of the normoglycemic mice tread with polydatin.

The effects of polydatin on salivation in normoglycemic mice as well as db/db mice were studied in this work, but one of the limitations of this study was that the animal study using the normoglycemic mice was a pilot study to determine the oral dose of polydatin. Therefore, there is not enough analysis data at the molecular level. Another limitation of this study was that multiple oral doses of polydatin were required to determine the effective dose range in the animal study using the db/db mice. The detailed beneficial role of polydatin on salivation under normoglycemic and hyperglycemic conditions needs to be studied further.

**Figure 7:**
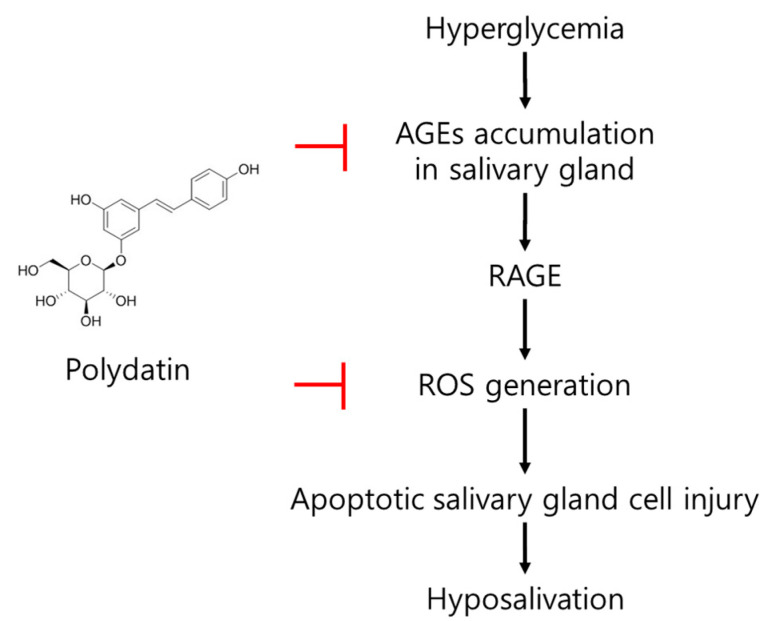
The possible mode of action of polydatin on diabetes-induced hypofunction of salivary glands.

In conclusion, an increase in ROS, a glycation product in the submandibular gland; a decrease in AQP5, which plays an important role in salvation caused by hyperglycemia, an apoptotic signal increase; and a salivation decrease in type 2 diabetic mice were confirmed. Polydatin mitigated the generation of excessive ROS due to the reduction of the glycation process initiated by the anti-glycation effect of polydatin and upregulated AQP5 expression, which is presumed to lead to the improvement of diabetic salivary gland dysfunction (Figure 7). Overall, this study shows polydatin’s potential for use as a therapeutic drug in diabetic-xerostomia due to its anti-glycation and antioxidant effects.

## Figures and Tables

**Table 1:** Body weight and blood glucose.

	Normal	DM	DM + PD
Body weight (g)	26.46 ± 0.37	30.74 ± 1.30 *	30.06 ± 1.86 *
Blood glucose (mg/dL)	179.29 ± 17.61	571.33 ± 70.22 *	562.86 ± 98.27

All data are expressed as the mean ± the SD. * *p* < 0.05 vs. normal control group.

## Data Availability

The data presented in this study are available on request from the corresponding author.

## References

[1] Miranda-Rius J., Brunet-Llobet L., Lahor-Soler E., Farré M. (2015). Salivary secretory disorders, inducing drugs, and clinical management. Int. J. Med. Sci..

[2] Proctor G.B. (2016). The physiology of salivary secretion. Periodontology 2000.

[3] Mese H., Matsuo R. (2007). Salivary secretion, taste and hyposalivation. J. Oral Rehabil..

[4] van der Putten G.J., Brand H.S., Schols J.M., de Baat C. (2011). The diagnostic suitability of a xerostomia questionnaire and the association between xerostomia, hyposalivation and medication use in a group of nursing home residents. Clin. Oral Investig..

[5] Guggenheimer J., Moore P.A. (2003). Xerostomia: Etiology, recognition and treatment. J. Am. Dent. Assoc..

[6] Atkinson J.C., Grisius M., Massey W. (2005). Salivary hypofunction and xerostomia: Diagnosis and treatment. Dent. Clin. N. Am..

[7] Moore P.A., Guggenheimer J., Etzel K.R., Weyant R.J., Orchard T. (2001). Type 1 diabetes mellitus, xerostomia, and salivary flow rates. Oral Surg. Oral Med. Oral Pathol. Oral Radiol. Endod..

[8] Feng J.K., Lu Y.F., Li J., Qi Y.H., Yi M.L., Ma D.Y. (2015). Upregulation of salivary α2 macroglobulin in patients with type 2 diabetes mellitus. Genet. Mol. Res..

[9] Singh V.P., Bali A., Singh N., Jaggi A.S. (2014). Advanced glycation end products and diabetic complications. Korean J. Physiol. Pharmacol..

[10] Sreebny L.M., Yu A., Green A., Valdini A. (1992). Xerostomia in diabetes mellitus. Diabetes Care.

[11] Rohani B. (2019). Oral manifestations in patients with diabetes mellitus. World J. Diabetes.

[12] Cicmil S., Mladenović I., Krunić J., Ivanović D., Stojanovic N. (2018). Oral alterations in diabetes mdellitus. Balk. J. Dent. Med..

[13] Visvanathan V., Nix P. (2010). Managing the patient presenting with xerostomia: A review. Int. J. Clin. Pract..

[14] Bascones-Martínez A., Muñoz-Corcuera M., Bascones-Ilundain C. (2015). Side effects of drugs on the oral cavity. Med. Clin..

[15] Selvan S.R., Venugopalan S. (2013). Effect of oral hypoglycemic drugs on salivary flow—A review. Int. J. Pharmtech. Res..

[16] Xie X., Peng J., Huang K., Huang J., Shen X., Liu P., Huang H. (2012). Polydatin ameliorates experimental diabetes-induced fibronectin through inhibiting the activation of NF-κB signaling pathway in rat glomerular mesangial cells. Mol. Cell Endocrinol..

[17] Du Q.H., Peng C., Zhang H. (2013). Polydatin: A review of pharmacology and pharmacokinetics. Pharm. Biol..

[18] Wang Y., Ye J., Li J., Chen C., Huang J., Liu P., Huang H. (2016). Polydatin ameliorates lipid and glucose metabolism in type 2 diabetes mellitus by downregulating proprotein convertase subtilisin/kexin type 9 (PCSK9). Cardiovasc. Diabetol..

[19] Şimşek G., Gürocak S., Karadaǧ N., Karabulut A.B., Demirtaş E., Karataş E., Pepele E. (2012). Protective effects of resveratrol on salivary gland damage induced by total body irradiation in rats. Laryngoscope.

[20] Xu L., Yang X., Cai J., Ma J., Cheng H., Zhao K., Yang L., Cao Y., Qin Q., Zhang C. (2013). Resveratrol attenuates radiation-induced salivary gland dysfunction in mice. Laryngoscope.

[21] Inoue H., Kishimoto A., Ushikoshi-Nakayama R., Hasaka A., Takahashi A., Ryo K., Muramatsu T., Ide F., Mishima K., Saito I. (2016). Resveratrol improves salivary dysfunction in a non-obese diabetic (NOD) mouse model of Sjögren’s syndrome. J. Clin. Biochem. Nutr..

[22] Yan S.F., Ramasamy R., Schmidt A.M. (2008). Mechanisms of disease: Advanced glycation end-products and their receptor in inflammation and diabetes complications. Nat. Clin. Pract. Endocrinol. Metab..

[23] Yao D., Brownlee M. (2010). Hyperglycemia-induced reactive oxygen species increase expression of the receptor for advanced glycation end products (RAGE) and RAGE ligands. Diabetes.

[24] Tang D., Kang R., Zeh H.J., Lotze M.T. (2011). High-mobility group box 1, oxidative stress, and disease. Antioxid. Redox. Signal..

[25] Valavanidis A., Vlachogianni T., Fiotakis C. (2009). 8-hydroxy-2’ -deoxyguanosine (8-OHdG): A critical biomarker of oxidative stress and carcinogenesis. J. Environ. Sci. Health C Environ. Carcinog. Ecotoxicol. Rev..

[26] Xiang R.-L., Huang Y., Zhang Y., Cong X., Zhang Z.-J., Wu L.-L., Yu G.-Y. (2020). Type 2 diabetes-induced hyposalivation of the submandibular gland through PINK1/Parkin-mediated mitophagy. J. Cell. Physiol..

[27] Allushi B., Bagavant H., Papinska J., Deshmukh U.S. (2019). Hyperglycemia and salivary gland dysfunction in the non-obese diabetic mouse: Caveats for preclinical studies in Sjögren’s syndrome. Sci. Rep..

[28] Fedirko N.V., Kruglikov I.A., Kopach O.V., Vats J.A., Kostyuk P.G., Voitenko N.V. (2006). Changes in functioning of rat submandibular salivary gland under streptozotocin-induced diabetes are associated with alterations of Ca^2+^ signaling and Ca^2+^ transporting pumps. Biochim. Biophys. Acta.

[29] Wang L., Huang L., Li N., Miao J., Liu W., Yu J. (2019). Ameliorative effect of polydatin on hyperglycemia and renal injury in streptozotocin-induced diabetic rats. Cell Mol. Biol..

[30] Yousef A., Shawki H., El-Shahawy A., El-Twab S., Abdel Moneim A., Oishi H. (2021). Polydatin mitigates pancreatic β-cell damage through its antioxidant activity. Biomed. Pharmacother..

[31] Gong W., Li J., Chen Z., Huang J., Chen Q., Cai W., Liu P., Huang H. (2017). Polydatin promotes Nrf2-ARE anti-oxidative pathway through activating CKIP-1 to resist HG-induced up-regulation of FN and ICAM-1 in GMCs and diabetic mice kidneys. Free Radic. Biol. Med..

[32] Chen C., Huang K., Hao J., Huang J., Yang Z., Xiong F., Liu P., Huang H. (2016). Polydatin attenuates AGEs-induced upregulation of fibronectin and ICAM-1 in rat glomerular mesangial cells and db/db diabetic mice kidneys by inhibiting the activation of the SphK1-S1P signaling pathway. Mol. Cell Endocrinol..

[33] Zheng L., Wu J., Mo J., Guo L., Wu X., Bao Y. (2019). Polydatin inhibits adipose tissue inflammation and ameliorates lipid metabolism in high-fat-fed mice. Biomed Res. Int..

[34] Tramunt B., Smati S., Grandgeorge N., Lenfant F., Arnal J.F., Montagner A., Gourdy P. (2020). Sex differences in metabolic regulation and diabetes susceptibility. Diabetologia.

[35] Elzinga S.E., Savelieff M.G., O’Brien P.D., Mendelson F.E., Hayes J.M., Feldman E.L. (2021). Sex differences in insulin resistance, but not peripheral neuropathy, in a diet-induced prediabetes mouse model. Dis. Model Mech..

[36] Salsali A., Nathan M. (2006). A review of types 1 and 2 diabetes mellitus and their treatment with insulin. Am. J. Ther..

[37] Kang W.S., Jung W.K., Park S.-B., Kim H.R., Kim J. (2021). Gemigliptin suppresses salivary dysfunction in streptozotocin-induced diabetic rats. Biomed. Pharmacother..

[38] Du X.-L., Edelstein D., Rossetti L., Fantus I.G., Goldberg H., Ziyadeh F., Wu J., Brownlee M. (2000). Hyperglycemia-induced mitochondrial superoxide overproduction activates the hexosamine pathway and induces plasminogen activator inhibitor-1 expression by increasing Sp1 glycosylation. Proc. Natl. Acad. Sci. USA.

[39] Yan L.J. (2018). Redox imbalance stress in diabetes mellitus: Role of the polyol pathway. Anim. Model Exp. Med..

[40] Auten R.L., Davis J.M. (2009). Oxygen toxicity and reactive oxygen species: The devil is in the details. Pediatr. Res..

[41] Kranstuber A.L., Del Rio C., Biesiadecki B.J., Hamlin R.L., Ottobre J., Gyorke S., Lacombe V.A. (2012). Advanced glycation end product cross-link breaker attenuates diabetes-induced cardiac dysfunction by improving sarcoplasmic reticulum calcium handling. Front. Physiol..

[42] Sanajou D., Ghorbani Haghjo A., Argani H., Aslani S. (2018). AGE-RAGE axis blockade in diabetic nephropathy: Current status and future directions. Eur. J. Pharmacol..

[43] Negre-Salvayre A., Salvayre R., Augé N., Pamplona R., Portero-Otín M. (2009). Hyperglycemia and glycation in diabetic complications. Antioxid. Redox. Signal..

[44] Matsumoto N., Omagari D., Ushikoshi-Nakayama R., Yamazaki T., Inoue H., Saito I. (2021). Hyperglycemia induces generation of reactive oxygen species and accelerates apoptotic cell death in salivary gland cells. Pathobiology.

[45] Katz J., Stavropoulos F., Bhattacharyya I., Stewart C., Perez F.M., Caudle R.M. (2004). Receptor of advanced glycation end product (RAGE) expression in the minor salivary glands of patients with Sjögren’s syndrome: A preliminary study. Scand. J. Rheumatol..

[46] Maleki V., Foroumandi E., Hajizadeh-Sharafabad F., Kheirouri S., Alizadeh M. (2020). The effect of resveratrol on advanced glycation end products in diabetes mellitus: A systematic review. Arch. Physiol. Biochem..

[47] Sheng Z., Ai B., Zheng L., Zheng X., Xu Z., Shen Y., Jin Z. (2018). Inhibitory activities of kaempferol, galangin, carnosic acid and polydatin against glycation and α-amylase and α-glucosidase enzymes. Int. J. Food Sci. Technol..

[48] Chen L., Chen Z., Xu Z., Feng W., Yang X., Qi Z. (2021). Polydatin protects Schwann cells from methylglyoxal induced cytotoxicity and promotes crushed sciatic nerves regeneration of diabetic rats. Phytother. Res..

[49] Yu Y., Tang D., Kang R. (2015). Oxidative stress-mediated HMGB1 biology. Front. Physiol..

[50] Prasad R., Liu Y., Deterding L.J., Poltoratsky V.P., Kedar P.S., Horton J.K., Kanno S., Asagoshi K., Hou E.W., Khodyreva S.N. (2007). HMGB1 is a cofactor in mammalian base excision repair. Mol. Cell.

[51] Kim C.S., Jo K., Kim J.S., Pyo M.K., Kim J. (2017). GS-E3D, a new pectin lyase-modified red ginseng extract, inhibited diabetes-related renal dysfunction in streptozotocin-induced diabetic rats. BMC Complement. Altern. Med..

[52] Fukuoka C.Y., Simões A., Uchiyama T., Arana-Chavez V.E., Abiko Y., Kuboyama N., Bhawal U.K. (2017). The effects of low-power laser irradiation on inflammation and apoptosis in submandibular glands of diabetes-induced rats. PLoS ONE.

[53] Ryo K., Yamada H., Nakagawa Y., Tai Y., Obara K., Inoue H., Mishima K., Saito I. (2006). Possible involvement of oxidative stress in salivary gland of patients with Sjogren’s syndrome. Pathobiology.

[54] Martín A.R., Villegas I., La Casa C., de la Lastra C.A. (2004). Resveratrol, a polyphenol found in grapes, suppresses oxidative damage and stimulates apoptosis during early colonic inflammation in rats. Biochem. Pharmacol..

[55] Wang H.L., Gao J.P., Han Y.L., Xu X., Wu R., Gao Y., Cui X.H. (2015). Comparative studies of polydatin and resveratrol on mutual transformation and antioxidative effect in vivo. Phytomedicine.

[56] Regev-Shoshani G., Shoseyov O., Bilkis I., Kerem Z. (2003). Glycosylation of resveratrol protects it from enzymic oxidation. Biochem. J..

[57] Perrella F., Coppola F., Petrone A., Platella C., Montesarchio D., Stringaro A., Ravagnan G., Fuggetta M.P., Rega N., Musumeci D. (2021). Interference of polydatin/resveratrol in the ACE2: Spike recognition during COVID-19 infection. A focus on their potential mechanism of action through computational and biochemical assays. Biomolecules.

[58] Huang Y., Mao Q.Y., Shi X.J., Cong X., Zhang Y., Wu L.L., Yu G.Y., Xiang R.L. (2020). Disruption of tight junctions contributes to hyposalivation of salivary glands in a mouse model of type 2 diabetes. J. Anat..

[59] Castro I., Barrera M.-J., González S., Aguilera S., Urzúa U., Cortés J., González M.-J. (2017). Mucins in salivary gland development, regeneration, and disease. Salivary Gland Development and Regeneration: Advances in Research and Clinical Approaches to Functional Restoration.

[60] Maciejczyk M., Skutnik-Radziszewska A., Zieniewska I., Matczuk J., Domel E., Waszkiel D., Żendzian-Piotrowska M., Szarmach I., Zalewska A. (2019). Antioxidant defense, oxidative modification, and salivary gland function in an early phase of cerulein pancreatitis. Oxid. Med. Cell. Longev..

[61] Ambudkar I.S. (2014). Ca^2+^ signaling and regulation of fluid secretion in salivary gland acinar cells. Cell Calcium.

[62] Agre P., King L.S., Yasui M., Guggino W.B., Ottersen O.P., Fujiyoshi Y., Engel A., Nielsen S. (2002). Aquaporin water channels--from atomic structure to clinical medicine. J. Physiol..

[63] Satoh K., Narita T., Matsuki-Fukushima M., Okabayashi K., Ito T., Senpuku H., Sugiya H. (2013). E2f1-deficient NOD/SCID mice have dry mouth due to a change of acinar/duct structure and the down-regulation of AQP5 in the salivary gland. Pflugers Arch..

[64] Ma T., Song Y., Gillespie A., Carlson E.J., Epstein C.J., Verkman A.S. (1999). Defective secretion of saliva in transgenic mice lacking aquaporin-5 water channels. J. Biol. Chem..

[65] Saito K., Mori S., Date F., Hong G. (2015). Epigallocatechin gallate stimulates the neuroreactive salivary secretomotor system in autoimmune sialadenitis of MRL-Fas(lpr) mice via activation of cAMP-dependent protein kinase A and inactivation of nuclear factor κB. Autoimmunity.

[66] Di Benedetto A., Posa F., De Maria S., Ravagnan G., Ballini A., Porro C., Trotta T., Grano M., Muzio L.L., Mori G. (2018). Polydatin, natural precursor of resveratrol, promotes osteogenic differentiation of mesenchymal stem cells. Int. J. Med. Sci..

[67] Huang H., Tindall D.J. (2007). Dynamic FoxO transcription factors. J. Cell Sci..

[68] Motta M.C., Divecha N., Lemieux M., Kamel C., Chen D., Gu W., Bultsma Y., McBurney M., Guarente L. (2004). Mammalian SIRT1 represses forkhead transcription factors. Cell.

[69] Lee S.M., Lee S.W., Kang M., Choi J.K., Park K., Byun J.S., Kim D.Y. (2021). FoxO1 as a Regulator of Aquaporin 5 Expression in the Salivary Gland. J. Dent. Res..

[70] Matsuzaki T., Suzuki T., Koyama H., Tanaka S., Takata K. (1999). Aquaporin-5 (AQP5), a water channel protein, in the rat salivary and lacrimal glands: Immunolocalization and effect of secretory stimulation. Cell Tissue Res..

